# Crystal structure of 2-acetyl-5-(3,4-di­meth­oxy­phen­yl)-6-eth­oxy­carbonyl-3,7-dimethyl-5*H*-thia­zolo[3,2-*a*]pyrimidin-8-ium chloride

**DOI:** 10.1107/S2056989015016229

**Published:** 2015-09-17

**Authors:** N. L. Prasad, M. S. Krishnamurthy, Noor Shahina Begum

**Affiliations:** a Department of Studies in Chemistry, Central College Campus, Bangalore University, Bangalore 560 001, Karnataka, India

**Keywords:** crystal structure, salt, pyrimidinium, chloride, pyrimidine derivatives, pharmacological properties, biological activity, hydrogen bonding, C—H⋯π inter­actions

## Abstract

The title mol­ecular salt, C_21_H_25_N_2_O_5_S^+^·Cl^−^, crystallizes with two ion pairs in the asymmetric unit. The cations have similar conformations (r.m.s. overlay fit = 0.40 Å), with one of them showing disorder of the terminal methyl group of the ester in a 0.72 (2):0.28 (2) ratio. In the first cation, the 3,4-dimeth­oxy-substituted phenyl ring subtends a dihedral angle of 88.38 (7)° with the pyrimidine ring and 6.79 (8)° with the thia­zole ring. The equivalent data for the second cation are 89.97 (3) and 6.42 (7)°, respectively. The pyrimidine ring adopts a sofa conformation in each cation. In the crystal, the components are linked by N—H⋯Cl hydrogen bonds, generating isolated ion pairs. The ion pairs are are linked by C—H⋯O inter­actions, generating a three-dimensional network. In addition, a weak C—H⋯π inter­action is observed.

## Related literature   

For the pharmacological properties of pyrimidine derivatives, see: Ashok *et al.* (2007 ▸); Alam *et al.* (2010 ▸); Kulakov *et al.* (2009 ▸); Zhi *et al.* (2008 ▸). For conformational effects on biological activity, see: Rovnyak *et al.* (1995 ▸). For related structures, see: Prasad *et al.* (2014 ▸); Nagarajaiah *et al.* (2012 ▸).
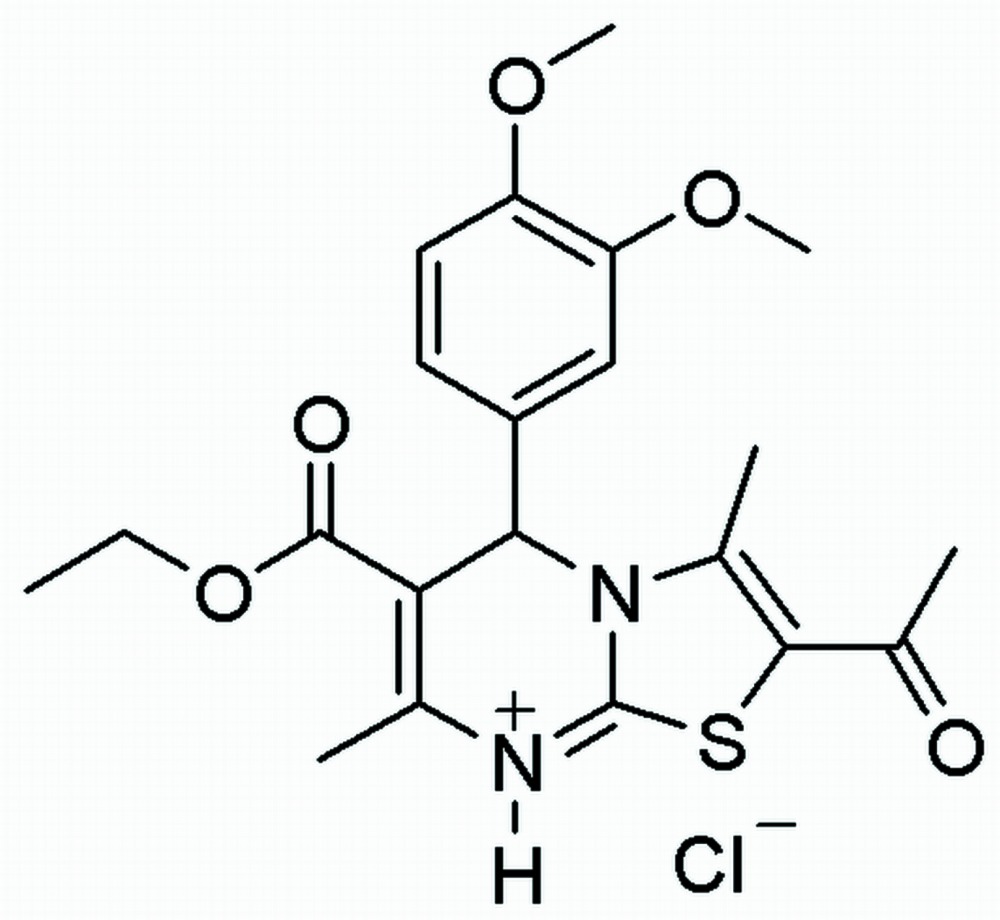


## Experimental   

### Crystal data  

C_21_H_25_N_2_O_5_S^+^·Cl^−^
*M* *_r_* = 452.94
Triclinic, 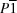

*a* = 9.9000 (6) Å
*b* = 11.8563 (7) Å
*c* = 19.0377 (11) Å
α = 80.827 (2)°
β = 83.999 (2)°
γ = 86.071 (2)°
*V* = 2190.9 (2) Å^3^
*Z* = 4
Mo *K*α radiation
μ = 0.31 mm^−1^
*T* = 100 K
0.40 × 0.35 × 0.30 mm

### Data collection  

Bruker SMART APEX CCD diffractometer
Absorption correction: multi-scan (*SADABS*; Bruker, 1998 ▸) *T*_min_ = 0.947, *T*_max_ = 0.953
26782 measured reflections
7693 independent reflections
4545 reflections with *I* > 2σ(*I*)
*R*_int_ = 0.077

### Refinement  

*R*[*F* ^2^ > 2σ(*F* ^2^)] = 0.055
*wR*(*F* ^2^) = 0.134
*S* = 0.97
7693 reflections
565 parameters
H-atom parameters constrained
Δρ_max_ = 0.46 e Å^−3^
Δρ_min_ = −0.39 e Å^−3^

### 

Data collection: *SMART* (Bruker, 1998 ▸); cell refinement: *SAINT-Plus* (Bruker, 1998 ▸); data reduction: *SAINT-Plus*; program(s) used to solve structure: *SHELXS97* (Sheldrick, 2008 ▸); program(s) used to refine structure: *SHELXL97* (Sheldrick, 2008 ▸); molecular graphics: *ORTEP-3 for Windows* (Farrugia, 2012 ▸) and *CAMERON* (Watkin *et al.*, 1996 ▸); software used to prepare material for publication: *WinGX* (Farrugia, 2012 ▸).

## Supplementary Material

Crystal structure: contains datablock(s) global, I. DOI: 10.1107/S2056989015016229/hb7484sup1.cif

Structure factors: contains datablock(s) I. DOI: 10.1107/S2056989015016229/hb7484Isup2.hkl

Supporting information file. DOI: 10.1107/S2056989015016229/hb7484Isup3.cml

**Fig. 1.**
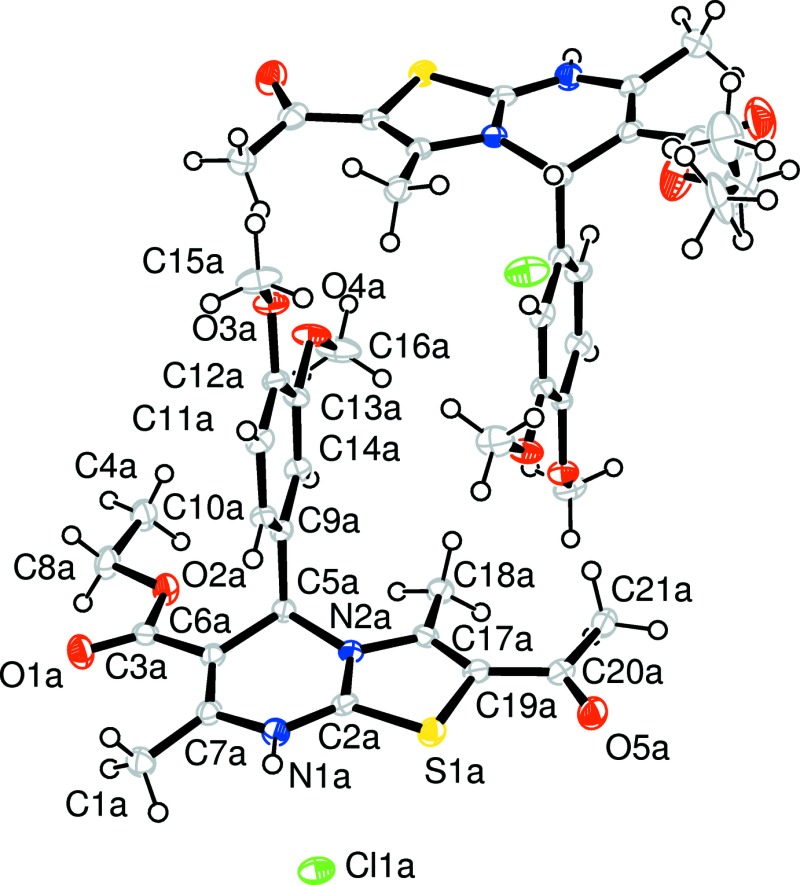
The molecular structure of the title compound with displacement ellipsoids drawn at the 50% probability level.

. DOI: 10.1107/S2056989015016229/hb7484fig1.tif
The mol­ecular structure of the title compound with displacement ellipsoids drawn at the 50% probability level.

**Fig. 2.**
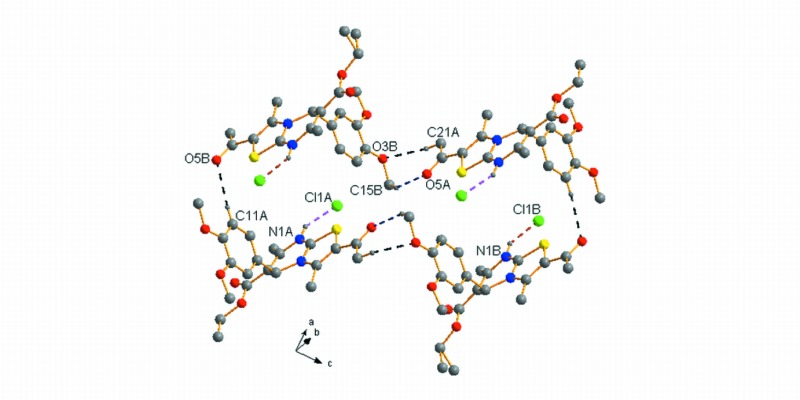
Unit-cell packing of the title compound showing C—H···O and N—H···Cl interactions with dotted lines. H-atoms not involved in hydrogen bonding have been excluded.

. DOI: 10.1107/S2056989015016229/hb7484fig2.tif
Unit-cell packing of the title compound showing C—H⋯O and N—H⋯Cl inter­actions with dotted lines. H-atoms not involved in hydrogen bonding have been excluded.

**Fig. 3.**
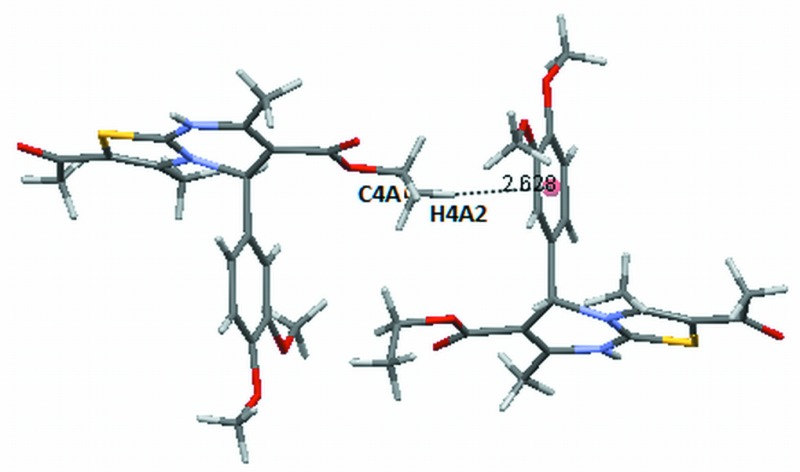
Unit-cell packing depicting the intermolecular C—H···π interactions with dotted lines.

. DOI: 10.1107/S2056989015016229/hb7484fig3.tif
Unit-cell packing depicting the inter­molecular C—H⋯π inter­actions with dotted lines.

CCDC reference: 1421372

Additional supporting information:  crystallographic information; 3D view; checkCIF report

## Figures and Tables

**Table 1:** Hydrogen-bond geometry (, ) *Cg*3 is the centroid of the C9*B*C14*B* ring.

*D*H*A*	*D*H	H*A*	*D* *A*	*D*H*A*
N1*A*H1*A*Cl1*A*	0.88	2.20	2.993(1)	151
N1*B*H1*B*Cl1*B* ^i^	0.88	2.20	2.992(2)	150
C11*A*H11*A*O5*B* ^ii^	0.95	2.58	3.523(1)	173
C15*B*H15*C*O5*A* ^iii^	0.98	2.58	3.456(6)	148
C21*A*H21*D*O3*B* ^iii^	0.98	2.50	3.459(6)	166
C4*A*H4*A*2*Cg*3	0.98	2.63	3.551(2)	157

Symmetry codes: (i) 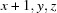; (ii) 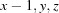; (iii) 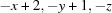.

## supplementary crystallographic information

### S1. Comment

Pyrimidine derivatives are of interest because of their useful biological and therapeutic activities (Ashok *et al.*, 2007). The presence of both pyrimidine and thiazole rings results in enhanced activity (Alam *et al.*, 2010; Kulakov *et al.*, 2009). Thiazolo [3,2-*a*]pyrimidine derivatives generate enzyme inhibitors as novel therapeutical entities for severe neurodegenerative diseases (Zhi *et al.*, 2008). In continuation to our research interests on thiazolo[3,2-*a*]pyrimidine derivatives (Prasad *et al.*, 2014; Nagarajaiah *et al.*, 2012), we report the crystal structure of the title compound. During the synthesis of one of the thiazolopyrimidine derivatives, an intermediate [C_21_H_25_N_2_O_5_S]^+^ Cl^-^ (I) was isolated and the structure was confirmed by X-ray diffraction.

The molecular structure of the compound is shown in Fig. 1. The title compound, [C_21_H_25_N_2_O_5_S]^+^ Cl^-^, crystallizes in the triclinic space group with two molecules in the asymmetric unit. In molecules A and B, the 3,4- dimethoxy substituted phenyl ring form dihedral angle of 88.38 (7)° / 89.97 (3)° with mean plane of the pyrimidine ring and 6.79 (8)° / 6.42 (7)° with thiazole ring respectively. The two values separated by / corresponds to the two molecules in the asymmetric unit. The pyrimidine ring adopts a *sofa* conformation with atom C5A displaced by -0.2637 (3) Å from the mean plane of the other five atoms (N1A/C2A/N2A/C6A/C7A). The carbonyl group of the exocyclic ester at C6A and C6B adopts a *cis* orientation with respect to C6A=C7A and C6B=C6B double bond and the 3,4-dimethoxy substituted phenyl ring shows an *syn* periplanar conformation with respect to C5A—H5A and C5B—H5B bond of the pyrimidine ring. Phenyl ring at C5A and C5B in both the molecules shows antagonist (aryl-group up) conformation (Rovnyak *et al.*, 1995). The packing features C—H···O interactions. The weak C11A—H11A···O5B hydrogen bonds forms supramolecular assembly along the crystallographic [101], which are inturn linked by another two C—H···O interactions (C15B—H15C···O5A & C21A—H21D···O3B) resulting in a ring with the graph set *R*^2^_2_(7) notation (Table. 1; Fig. 2). The crystal packing also features N1A—H1A···Cl1A & N1B—H1B···Cl1B interactions. In addition, a weak C—H···π interaction of the type C4A-H4A2···*Cg* (*Cg* being the centroid of the phenyl ring) at a distance of 2.628 Å is also observed (Fig. 3).

### S2. Experimental

A mixture of 4-(3,4-dimethoxy-phenyl)-6-methyl-2-thioxo-1,2,3,4-tetrahydro- pyrimidine-5-carboxylic acid ethyl ester (10 mmol) and 3-chloro-2,4- pentanedione (10 mmol) was refluxed in dry ethanol (20 mmol) for 12 h. The excess of solvent was distilled off and the solid hydrochloride salt that separated was collected by filtration, suspended in water and neutralized by aqueous sodium carbonate solution to yield the free base. The solution was filtered, the solid washed with water, dried and recrystallized from ethyl acetate to give the title compound (74% yield, mp 385 K). The compound was recrystallized by slow evaporation from 1:1 mixture of ethyl acetate and methanol, yielding pale yellow blocks.

### S3. Refinement

The H atoms were placed at calculated positions in the riding-model approximation with C—H = 0.95° A, 1.00 ° A and 0.96 ° A for aromatic, methyne and methyl H-atoms respectively, with *U*_iso_(H) = 1.5*U*_eq_(C) for methyl H atoms and *U*_iso_(H) = 1.2Ueq(C) for other hydrogen atoms.

### Crystal data

**Table:**

C_21_H_25_N_2_O_5_S^+^·Cl^−^	*Z* = 4
*M**_r_* = 452.94	*F*(000) = 952
Triclinic, *P*1	*D*_x_ = 1.373 Mg m^−^^3^
Hall symbol: -P 1	Mo *K*α radiation, λ = 0.71073 Å
*a* = 9.9000 (6) Å	Cell parameters from 7693 reflections
*b* = 11.8563 (7) Å	θ = 2.1–25.0°
*c* = 19.0377 (11) Å	µ = 0.31 mm^−^^1^
α = 80.827 (2)°	*T* = 100 K
β = 83.999 (2)°	Block, colorless
γ = 86.071 (2)°	0.40 × 0.35 × 0.30 mm
*V* = 2190.9 (2) Å^3^	

### Data collection

**Table:**

Bruker SMART APEX CCD diffractometer	7693 independent reflections
Radiation source: fine-focus sealed tube	4545 reflections with *I* > 2σ(*I*)
Graphite monochromator	*R*_int_ = 0.077
ω scans	θ_max_ = 25.0°, θ_min_ = 2.1°
Absorption correction: multi-scan (*SADABS*; Bruker, 1998)	*h* = −11→11
*T*_min_ = 0.947, *T*_max_ = 0.953	*k* = −14→14
26782 measured reflections	*l* = −22→22

### Refinement

**Table:**

Refinement on *F*^2^	Primary atom site location: structure-invariant direct methods
Least-squares matrix: full	Secondary atom site location: difference Fourier map
*R*[*F*^2^ > 2σ(*F*^2^)] = 0.055	Hydrogen site location: inferred from neighbouring sites
*wR*(*F*^2^) = 0.134	H-atom parameters constrained
*S* = 0.97	*w* = 1/[σ^2^(*F*_o_^2^) + (0.0651*P*)^2^] where *P* = (*F*_o_^2^ + 2*F*_c_^2^)/3
7693 reflections	(Δ/σ)_max_ = 0.001
565 parameters	Δρ_max_ = 0.46 e Å^−^^3^
0 restraints	Δρ_min_ = −0.39 e Å^−^^3^

### Special details

**Table:**

Geometry. All s.u.'s (except the s.u. in the dihedral angle between two l.s. planes) are estimated using the full covariance matrix. The cell s.u.'s are taken into account individually in the estimation of s.u.'s in distances, angles and torsion angles; correlations between s.u.'s in cell parameters are only used when they are defined by crystal symmetry. An approximate (isotropic) treatment of cell s.u.'s is used for estimating s.u.'s involving l.s. planes.
Refinement. Refinement of *F*^2^ against ALL reflections. The weighted *R*-factor *wR* and goodness of fit *S* are based on *F*^2^, conventional *R*-factors *R* are based on *F*, with *F* set to zero for negative *F*^2^. The threshold expression of *F*^2^ > 2σ(*F*^2^) is used only for calculating *R*-factors(gt) *etc*. and is not relevant to the choice of reflections for refinement. *R*-factors based on *F*^2^ are statistically about twice as large as those based on *F*, and *R*- factors based on ALL data will be even larger.

### Fractional atomic coordinates and isotropic or equivalent isotropic displacement parameters (Å^2^)

**Table:**

	*x*	*y*	*z*	*U*_iso_*/*U*_eq_	Occ. (<1)
S1A	0.55372 (9)	0.82102 (7)	0.06988 (5)	0.0231 (2)	
S1B	1.58661 (9)	0.56682 (7)	0.41046 (5)	0.0236 (2)	
Cl1A	0.26054 (8)	0.98395 (7)	0.07281 (4)	0.0258 (2)	
O3B	1.1844 (2)	0.61890 (18)	0.07522 (11)	0.0219 (6)	
O3A	0.9580 (2)	0.78589 (18)	0.40798 (11)	0.0233 (6)	
N1A	0.5318 (3)	1.0231 (2)	0.11824 (14)	0.0216 (7)	
H1A	0.4428	1.0205	0.1199	0.026*	
N2A	0.7460 (3)	0.9302 (2)	0.10151 (13)	0.0183 (7)	
O4A	1.1459 (2)	0.82817 (19)	0.30585 (12)	0.0274 (6)	
O4B	0.9931 (2)	0.58909 (18)	0.17602 (11)	0.0248 (6)	
O2A	0.9189 (2)	1.20032 (18)	0.15574 (12)	0.0255 (6)	
N2B	1.3831 (3)	0.4659 (2)	0.38338 (13)	0.0183 (6)	
C11A	0.7792 (3)	0.8628 (3)	0.33236 (17)	0.0200 (8)	
H11A	0.7091	0.8437	0.3696	0.024*	
O5A	0.6313 (2)	0.61710 (19)	0.01754 (13)	0.0312 (6)	
N1B	1.5897 (3)	0.3652 (2)	0.36194 (14)	0.0243 (7)	
H1B	1.6789	0.3658	0.3554	0.029*	
C9A	0.8485 (3)	0.9473 (3)	0.21133 (16)	0.0165 (8)	
O5B	1.5264 (2)	0.7675 (2)	0.46921 (13)	0.0330 (6)	
C14A	0.9846 (3)	0.9182 (3)	0.22318 (17)	0.0192 (8)	
H14A	1.0548	0.9376	0.1861	0.023*	
O1A	0.7242 (2)	1.2983 (2)	0.18268 (13)	0.0312 (6)	
C11B	1.3560 (3)	0.5287 (3)	0.15165 (17)	0.0211 (8)	
H11B	1.4278	0.5424	0.1146	0.025*	
C13B	1.1196 (3)	0.5473 (3)	0.19403 (17)	0.0188 (8)	
C10A	0.7475 (3)	0.9186 (3)	0.26544 (17)	0.0200 (8)	
H10A	0.6550	0.9371	0.2571	0.024*	
C2B	1.5186 (3)	0.4558 (3)	0.38183 (17)	0.0220 (8)	
C17B	1.3250 (3)	0.5635 (3)	0.40926 (16)	0.0197 (8)	
C5A	0.8131 (3)	1.0096 (3)	0.13895 (17)	0.0174 (8)	
H5A	0.8993	1.0330	0.1097	0.021*	
C9B	1.2801 (3)	0.4514 (3)	0.27319 (17)	0.0181 (8)	
C19B	1.4225 (3)	0.6284 (3)	0.42452 (17)	0.0204 (8)	
C6A	0.7208 (3)	1.1153 (3)	0.14404 (16)	0.0185 (8)	
C2A	0.6109 (3)	0.9356 (3)	0.09945 (16)	0.0185 (8)	
C21B	1.2888 (3)	0.8079 (3)	0.46114 (18)	0.0283 (9)	
H21A	1.2298	0.7711	0.5017	0.042*	
H21B	1.2430	0.8144	0.4174	0.042*	
H21C	1.3081	0.8844	0.4695	0.042*	
C14B	1.1472 (3)	0.4909 (3)	0.26048 (17)	0.0196 (8)	
H14B	1.0757	0.4789	0.2978	0.024*	
C12A	0.9138 (3)	0.8359 (3)	0.34386 (17)	0.0197 (8)	
C13A	1.0166 (3)	0.8614 (3)	0.28869 (17)	0.0184 (8)	
C12B	1.2245 (3)	0.5658 (3)	0.13895 (17)	0.0178 (8)	
C17A	0.8121 (3)	0.8373 (3)	0.07323 (17)	0.0197 (8)	
C20A	0.7354 (4)	0.6582 (3)	0.02851 (17)	0.0237 (8)	
C7A	0.5872 (3)	1.1187 (3)	0.13521 (17)	0.0200 (8)	
C10B	1.3838 (3)	0.4711 (3)	0.21867 (17)	0.0220 (8)	
H10B	1.4745	0.4452	0.2270	0.026*	
C5B	1.3078 (3)	0.3898 (3)	0.34687 (17)	0.0204 (8)	
H5B	1.2187	0.3740	0.3755	0.025*	
C20B	1.4185 (4)	0.7379 (3)	0.45335 (17)	0.0257 (9)	
C3A	0.7829 (3)	1.2151 (3)	0.16276 (17)	0.0202 (8)	
C6B	1.3908 (4)	0.2779 (3)	0.34601 (17)	0.0218 (8)	
C7B	1.5250 (4)	0.2687 (3)	0.35115 (17)	0.0252 (9)	
C1A	0.4850 (3)	1.2162 (3)	0.14002 (19)	0.0277 (9)	
H1A1	0.4466	1.2143	0.1897	0.042*	
H1A2	0.4122	1.2100	0.1100	0.042*	
H1A3	0.5291	1.2885	0.1235	0.042*	
C19A	0.7212 (3)	0.7669 (3)	0.05676 (17)	0.0210 (8)	
C16B	0.8851 (3)	0.5858 (3)	0.23198 (18)	0.0323 (10)	
H16A	0.8678	0.5061	0.2517	0.048*	
H16B	0.8027	0.6239	0.2129	0.048*	
H16C	0.9106	0.6253	0.2697	0.048*	
C18A	0.9630 (3)	0.8299 (3)	0.06129 (17)	0.0222 (8)	
H18A	0.9998	0.7761	0.1001	0.033*	
H18B	0.9977	0.9056	0.0603	0.033*	
H18C	0.9908	0.8030	0.0156	0.033*	
C15B	1.2870 (3)	0.6565 (3)	0.01999 (18)	0.0291 (9)	
H15A	1.3409	0.7123	0.0359	0.044*	
H15B	1.2443	0.6921	−0.0229	0.044*	
H15C	1.3464	0.5908	0.0089	0.044*	
C1B	1.6186 (4)	0.1652 (3)	0.34882 (19)	0.0339 (10)	
H1B1	1.5867	0.1038	0.3864	0.051*	
H1B2	1.7105	0.1836	0.3565	0.051*	
H1B3	1.6202	0.1403	0.3021	0.051*	
C18B	1.1749 (3)	0.5825 (3)	0.41897 (18)	0.0243 (9)	
H18D	1.1493	0.6179	0.4620	0.036*	
H18E	1.1323	0.5091	0.4242	0.036*	
H18F	1.1439	0.6332	0.3772	0.036*	
C15A	0.8599 (4)	0.7449 (3)	0.46416 (18)	0.0349 (10)	
H15D	0.7958	0.8080	0.4749	0.052*	
H15E	0.9056	0.7135	0.5068	0.052*	
H15F	0.8105	0.6849	0.4496	0.052*	
O2B	1.1810 (3)	0.1986 (2)	0.35168 (14)	0.0473 (8)	
O1B	1.3641 (3)	0.0864 (2)	0.32700 (16)	0.0561 (9)	
C16A	1.2520 (3)	0.8421 (3)	0.24971 (19)	0.0390 (11)	
H16D	1.2328	0.8007	0.2116	0.058*	
H16E	1.3383	0.8118	0.2680	0.058*	
H16F	1.2584	0.9236	0.2307	0.058*	
C21A	0.8716 (3)	0.6000 (3)	0.01681 (19)	0.0322 (10)	
H21D	0.8623	0.5290	−0.0020	0.048*	
H21E	0.9125	0.5824	0.0622	0.048*	
H21F	0.9298	0.6504	−0.0176	0.048*	
C8A	0.9939 (3)	1.2897 (3)	0.17477 (19)	0.0288 (9)	
H8A1	0.9670	1.3005	0.2249	0.035*	
H8A2	0.9765	1.3631	0.1432	0.035*	
C4A	1.1415 (3)	1.2505 (3)	0.16523 (19)	0.0299 (9)	
H4A1	1.1584	1.1814	0.1996	0.045*	
H4A2	1.1980	1.3110	0.1734	0.045*	
H4A3	1.1642	1.2336	0.1165	0.045*	
C3B	1.3152 (4)	0.1763 (3)	0.34012 (19)	0.0322 (10)	
C8B	1.0940 (5)	0.1052 (4)	0.3517 (3)	0.0723 (16)	
H8B1	1.0246	0.1296	0.3178	0.087*	
H8B2	1.1491	0.0396	0.3356	0.087*	
C4B	1.0291 (12)	0.0708 (9)	0.4207 (6)	0.053 (3)	0.72 (2)
H4B1	0.9833	0.1376	0.4390	0.080*	0.72 (2)
H4B2	1.0969	0.0359	0.4527	0.080*	0.72 (2)
H4B3	0.9619	0.0149	0.4182	0.080*	0.72 (2)
C4B'	0.975 (2)	0.1130 (18)	0.3817 (19)	0.045 (8)	0.28 (2)
H4B4	0.9286	0.0432	0.3801	0.068*	0.28 (2)
H4B5	0.9250	0.1790	0.3567	0.068*	0.28 (2)
H4B6	0.9785	0.1231	0.4315	0.068*	0.28 (2)
Cl1B	0.87338 (9)	0.39271 (8)	0.39496 (5)	0.0349 (3)	

### Atomic displacement parameters (Å^2^)

**Table:**

	*U*^11^	*U*^22^	*U*^33^	*U*^12^	*U*^13^	*U*^23^
S1A	0.0175 (5)	0.0254 (5)	0.0285 (5)	−0.0026 (4)	−0.0046 (4)	−0.0088 (4)
S1B	0.0202 (5)	0.0268 (5)	0.0254 (5)	−0.0032 (4)	−0.0064 (4)	−0.0050 (4)
Cl1A	0.0188 (5)	0.0344 (6)	0.0240 (5)	−0.0049 (4)	−0.0049 (4)	−0.0001 (4)
O3B	0.0190 (13)	0.0284 (14)	0.0164 (13)	−0.0025 (11)	0.0002 (11)	0.0012 (11)
O3A	0.0181 (13)	0.0334 (15)	0.0160 (13)	−0.0020 (11)	−0.0025 (11)	0.0039 (11)
N1A	0.0111 (15)	0.0268 (18)	0.0286 (17)	0.0015 (13)	−0.0022 (13)	−0.0096 (14)
N2A	0.0165 (16)	0.0194 (16)	0.0196 (16)	0.0015 (13)	−0.0049 (13)	−0.0035 (13)
O4A	0.0138 (13)	0.0422 (16)	0.0225 (14)	0.0025 (11)	−0.0031 (12)	0.0056 (11)
O4B	0.0176 (14)	0.0328 (15)	0.0211 (13)	0.0024 (11)	−0.0013 (11)	0.0027 (11)
O2A	0.0155 (13)	0.0244 (14)	0.0400 (15)	−0.0045 (10)	−0.0040 (12)	−0.0127 (11)
N2B	0.0184 (17)	0.0186 (17)	0.0177 (16)	−0.0011 (13)	−0.0037 (13)	−0.0009 (13)
C11A	0.020 (2)	0.024 (2)	0.0153 (19)	−0.0058 (16)	0.0018 (16)	−0.0019 (15)
O5A	0.0253 (15)	0.0298 (15)	0.0424 (16)	−0.0015 (12)	−0.0096 (13)	−0.0130 (12)
N1B	0.0200 (17)	0.0266 (18)	0.0275 (17)	−0.0002 (14)	−0.0050 (14)	−0.0064 (14)
C9A	0.0164 (19)	0.0173 (19)	0.0161 (19)	−0.0024 (15)	−0.0009 (16)	−0.0036 (15)
O5B	0.0264 (15)	0.0367 (16)	0.0397 (16)	−0.0065 (12)	−0.0062 (13)	−0.0135 (13)
C14A	0.0156 (19)	0.022 (2)	0.020 (2)	−0.0028 (15)	−0.0014 (16)	−0.0027 (16)
O1A	0.0237 (14)	0.0276 (16)	0.0455 (17)	0.0033 (12)	−0.0050 (13)	−0.0161 (13)
C11B	0.022 (2)	0.023 (2)	0.018 (2)	−0.0075 (16)	0.0024 (16)	−0.0017 (16)
C13B	0.018 (2)	0.018 (2)	0.021 (2)	−0.0011 (15)	−0.0058 (17)	−0.0037 (15)
C10A	0.0190 (19)	0.021 (2)	0.022 (2)	0.0003 (15)	−0.0073 (17)	−0.0043 (16)
C2B	0.020 (2)	0.028 (2)	0.018 (2)	0.0010 (17)	−0.0063 (17)	−0.0013 (16)
C17B	0.025 (2)	0.021 (2)	0.0122 (18)	−0.0003 (16)	−0.0020 (16)	0.0006 (15)
C5A	0.0119 (18)	0.020 (2)	0.0215 (19)	−0.0001 (15)	−0.0024 (16)	−0.0062 (15)
C9B	0.021 (2)	0.019 (2)	0.0145 (19)	−0.0051 (15)	−0.0007 (16)	−0.0014 (15)
C19B	0.019 (2)	0.025 (2)	0.0164 (19)	−0.0001 (16)	−0.0058 (16)	−0.0009 (16)
C6A	0.023 (2)	0.018 (2)	0.0150 (18)	−0.0010 (15)	−0.0026 (16)	−0.0019 (15)
C2A	0.018 (2)	0.025 (2)	0.0139 (19)	−0.0012 (16)	−0.0033 (16)	−0.0035 (15)
C21B	0.031 (2)	0.028 (2)	0.026 (2)	0.0024 (18)	−0.0024 (18)	−0.0051 (17)
C14B	0.018 (2)	0.022 (2)	0.019 (2)	−0.0057 (15)	0.0015 (16)	−0.0055 (16)
C12A	0.024 (2)	0.019 (2)	0.017 (2)	−0.0028 (15)	−0.0078 (17)	−0.0004 (15)
C13A	0.0152 (19)	0.019 (2)	0.020 (2)	0.0012 (15)	−0.0021 (17)	−0.0009 (15)
C12B	0.025 (2)	0.0124 (19)	0.017 (2)	−0.0024 (15)	−0.0081 (17)	−0.0011 (15)
C17A	0.0191 (19)	0.020 (2)	0.0196 (19)	0.0037 (16)	−0.0041 (16)	−0.0025 (16)
C20A	0.026 (2)	0.026 (2)	0.020 (2)	−0.0009 (17)	−0.0056 (17)	−0.0050 (16)
C7A	0.024 (2)	0.018 (2)	0.0186 (19)	−0.0032 (16)	−0.0040 (17)	−0.0020 (15)
C10B	0.018 (2)	0.025 (2)	0.025 (2)	−0.0026 (16)	−0.0078 (17)	−0.0042 (16)
C5B	0.021 (2)	0.021 (2)	0.019 (2)	−0.0047 (16)	−0.0042 (16)	−0.0015 (15)
C20B	0.033 (2)	0.029 (2)	0.014 (2)	−0.0074 (18)	0.0012 (18)	0.0004 (16)
C3A	0.020 (2)	0.022 (2)	0.0182 (19)	0.0014 (16)	−0.0044 (17)	−0.0012 (16)
C6B	0.031 (2)	0.021 (2)	0.0134 (19)	−0.0059 (17)	−0.0053 (17)	−0.0001 (15)
C7B	0.035 (2)	0.026 (2)	0.0145 (19)	0.0016 (18)	−0.0071 (17)	−0.0030 (16)
C1A	0.022 (2)	0.028 (2)	0.035 (2)	0.0023 (17)	−0.0065 (18)	−0.0086 (18)
C19A	0.020 (2)	0.024 (2)	0.020 (2)	−0.0003 (16)	−0.0057 (16)	−0.0027 (16)
C16B	0.021 (2)	0.047 (3)	0.028 (2)	0.0037 (18)	0.0008 (19)	−0.0043 (19)
C18A	0.0162 (19)	0.026 (2)	0.025 (2)	0.0006 (15)	−0.0026 (16)	−0.0060 (16)
C15B	0.031 (2)	0.035 (2)	0.021 (2)	−0.0071 (18)	−0.0069 (18)	0.0009 (17)
C1B	0.041 (2)	0.029 (2)	0.032 (2)	0.0058 (19)	−0.011 (2)	−0.0054 (18)
C18B	0.021 (2)	0.029 (2)	0.025 (2)	0.0015 (16)	−0.0047 (17)	−0.0079 (17)
C15A	0.030 (2)	0.053 (3)	0.018 (2)	−0.010 (2)	−0.0044 (19)	0.0081 (18)
O2B	0.0406 (18)	0.0509 (19)	0.057 (2)	−0.0283 (15)	0.0087 (15)	−0.0242 (15)
O1B	0.062 (2)	0.0254 (18)	0.086 (2)	−0.0040 (15)	−0.0212 (18)	−0.0155 (16)
C16A	0.016 (2)	0.062 (3)	0.031 (2)	0.0072 (19)	−0.0007 (19)	0.010 (2)
C21A	0.031 (2)	0.032 (2)	0.040 (2)	0.0078 (18)	−0.015 (2)	−0.0190 (19)
C8A	0.027 (2)	0.025 (2)	0.038 (2)	−0.0073 (17)	−0.0041 (19)	−0.0133 (18)
C4A	0.022 (2)	0.029 (2)	0.041 (2)	−0.0012 (17)	−0.0060 (19)	−0.0137 (18)
C3B	0.048 (3)	0.028 (3)	0.021 (2)	−0.010 (2)	−0.009 (2)	0.0003 (18)
C8B	0.071 (4)	0.080 (4)	0.078 (4)	−0.057 (3)	0.011 (3)	−0.040 (3)
C4B	0.055 (6)	0.031 (5)	0.072 (7)	−0.016 (4)	0.007 (5)	−0.008 (4)
C4B'	0.034 (12)	0.013 (10)	0.09 (2)	−0.003 (8)	−0.003 (12)	−0.013 (10)
Cl1B	0.0262 (5)	0.0494 (7)	0.0274 (5)	−0.0088 (5)	−0.0059 (4)	0.0044 (5)

### Geometric parameters (Å, º)

**Table:**

S1A—C2A	1.702 (3)	C12A—C13A	1.396 (4)
S1A—C19A	1.744 (3)	C17A—C19A	1.361 (4)
S1B—C2B	1.706 (3)	C17A—C18A	1.485 (4)
S1B—C19B	1.747 (3)	C20A—C19A	1.466 (4)
O3B—C12B	1.360 (3)	C20A—C21A	1.484 (4)
O3B—C15B	1.423 (4)	C7A—C1A	1.491 (4)
O3A—C12A	1.371 (4)	C10B—H10B	0.9500
O3A—C15A	1.416 (4)	C5B—C6B	1.514 (4)
N1A—C2A	1.330 (4)	C5B—H5B	1.0000
N1A—C7A	1.389 (4)	C6B—C7B	1.339 (4)
N1A—H1A	0.8800	C6B—C3B	1.486 (5)
N2A—C2A	1.339 (4)	C7B—C1B	1.490 (4)
N2A—C17A	1.396 (4)	C1A—H1A1	0.9800
N2A—C5A	1.495 (4)	C1A—H1A2	0.9800
O4A—C13A	1.370 (4)	C1A—H1A3	0.9800
O4A—C16A	1.417 (4)	C16B—H16A	0.9800
O4B—C13B	1.372 (4)	C16B—H16B	0.9800
O4B—C16B	1.426 (4)	C16B—H16C	0.9800
O2A—C3A	1.341 (4)	C18A—H18A	0.9800
O2A—C8A	1.447 (3)	C18A—H18B	0.9800
N2B—C2B	1.336 (4)	C18A—H18C	0.9800
N2B—C17B	1.395 (4)	C15B—H15A	0.9800
N2B—C5B	1.501 (4)	C15B—H15B	0.9800
C11A—C12A	1.380 (4)	C15B—H15C	0.9800
C11A—C10A	1.395 (4)	C1B—H1B1	0.9800
C11A—H11A	0.9500	C1B—H1B2	0.9800
O5A—C20A	1.220 (4)	C1B—H1B3	0.9800
N1B—C2B	1.328 (4)	C18B—H18D	0.9800
N1B—C7B	1.401 (4)	C18B—H18E	0.9800
N1B—H1B	0.8800	C18B—H18F	0.9800
C9A—C10A	1.377 (4)	C15A—H15D	0.9800
C9A—C14A	1.400 (4)	C15A—H15E	0.9800
C9A—C5A	1.520 (4)	C15A—H15F	0.9800
O5B—C20B	1.229 (4)	O2B—C3B	1.339 (4)
C14A—C13A	1.376 (4)	O2B—C8B	1.449 (4)
C14A—H14A	0.9500	O1B—C3B	1.192 (4)
O1A—C3A	1.204 (4)	C16A—H16D	0.9800
C11B—C12B	1.379 (4)	C16A—H16E	0.9800
C11B—C10B	1.392 (4)	C16A—H16F	0.9800
C11B—H11B	0.9500	C21A—H21D	0.9800
C13B—C14B	1.378 (4)	C21A—H21E	0.9800
C13B—C12B	1.399 (4)	C21A—H21F	0.9800
C10A—H10A	0.9500	C8A—C4A	1.504 (4)
C17B—C19B	1.356 (4)	C8A—H8A1	0.9900
C17B—C18B	1.485 (4)	C8A—H8A2	0.9900
C5A—C6A	1.510 (4)	C4A—H4A1	0.9800
C5A—H5A	1.0000	C4A—H4A2	0.9800
C9B—C10B	1.385 (4)	C4A—H4A3	0.9800
C9B—C14B	1.399 (4)	C8B—C4B'	1.259 (16)
C9B—C5B	1.517 (4)	C8B—C4B	1.411 (9)
C19B—C20B	1.485 (4)	C8B—H8B1	0.9900
C6A—C7A	1.347 (4)	C8B—H8B2	0.9900
C6A—C3A	1.478 (4)	C4B—H4B1	0.9800
C21B—C20B	1.488 (4)	C4B—H4B2	0.9800
C21B—H21A	0.9800	C4B—H4B3	0.9800
C21B—H21B	0.9800	C4B'—H4B4	0.9800
C21B—H21C	0.9800	C4B'—H4B5	0.9800
C14B—H14B	0.9500	C4B'—H4B6	0.9800
			
C2A—S1A—C19A	89.49 (15)	O1A—C3A—C6A	126.9 (3)
C2B—S1B—C19B	88.95 (16)	O2A—C3A—C6A	110.1 (3)
C12B—O3B—C15B	118.0 (2)	C7B—C6B—C3B	120.8 (3)
C12A—O3A—C15A	118.4 (3)	C7B—C6B—C5B	122.6 (3)
C2A—N1A—C7A	121.1 (3)	C3B—C6B—C5B	116.5 (3)
C2A—N1A—H1A	119.5	C6B—C7B—N1B	118.5 (3)
C7A—N1A—H1A	119.5	C6B—C7B—C1B	128.0 (3)
C2A—N2A—C17A	113.5 (3)	N1B—C7B—C1B	113.5 (3)
C2A—N2A—C5A	121.4 (3)	C7A—C1A—H1A1	109.5
C17A—N2A—C5A	124.7 (3)	C7A—C1A—H1A2	109.5
C13A—O4A—C16A	117.1 (3)	H1A1—C1A—H1A2	109.5
C13B—O4B—C16B	117.6 (3)	C7A—C1A—H1A3	109.5
C3A—O2A—C8A	116.4 (3)	H1A1—C1A—H1A3	109.5
C2B—N2B—C17B	113.9 (3)	H1A2—C1A—H1A3	109.5
C2B—N2B—C5B	120.8 (3)	C17A—C19A—C20A	133.4 (3)
C17B—N2B—C5B	124.2 (3)	C17A—C19A—S1A	112.3 (3)
C12A—C11A—C10A	119.2 (3)	C20A—C19A—S1A	114.3 (2)
C12A—C11A—H11A	120.4	O4B—C16B—H16A	109.5
C10A—C11A—H11A	120.4	O4B—C16B—H16B	109.5
C2B—N1B—C7B	121.2 (3)	H16A—C16B—H16B	109.5
C2B—N1B—H1B	119.4	O4B—C16B—H16C	109.5
C7B—N1B—H1B	119.4	H16A—C16B—H16C	109.5
C10A—C9A—C14A	119.6 (3)	H16B—C16B—H16C	109.5
C10A—C9A—C5A	120.4 (3)	C17A—C18A—H18A	109.5
C14A—C9A—C5A	119.9 (3)	C17A—C18A—H18B	109.5
C13A—C14A—C9A	119.9 (3)	H18A—C18A—H18B	109.5
C13A—C14A—H14A	120.0	C17A—C18A—H18C	109.5
C9A—C14A—H14A	120.0	H18A—C18A—H18C	109.5
C12B—C11B—C10B	120.1 (3)	H18B—C18A—H18C	109.5
C12B—C11B—H11B	120.0	O3B—C15B—H15A	109.5
C10B—C11B—H11B	120.0	O3B—C15B—H15B	109.5
O4B—C13B—C14B	124.8 (3)	H15A—C15B—H15B	109.5
O4B—C13B—C12B	115.0 (3)	O3B—C15B—H15C	109.5
C14B—C13B—C12B	120.2 (3)	H15A—C15B—H15C	109.5
C9A—C10A—C11A	120.8 (3)	H15B—C15B—H15C	109.5
C9A—C10A—H10A	119.6	C7B—C1B—H1B1	109.5
C11A—C10A—H10A	119.6	C7B—C1B—H1B2	109.5
N1B—C2B—N2B	121.8 (3)	H1B1—C1B—H1B2	109.5
N1B—C2B—S1B	124.8 (3)	C7B—C1B—H1B3	109.5
N2B—C2B—S1B	113.4 (2)	H1B1—C1B—H1B3	109.5
C19B—C17B—N2B	110.8 (3)	H1B2—C1B—H1B3	109.5
C19B—C17B—C18B	128.9 (3)	C17B—C18B—H18D	109.5
N2B—C17B—C18B	120.3 (3)	C17B—C18B—H18E	109.5
N2A—C5A—C6A	109.0 (2)	H18D—C18B—H18E	109.5
N2A—C5A—C9A	109.4 (2)	C17B—C18B—H18F	109.5
C6A—C5A—C9A	113.1 (3)	H18D—C18B—H18F	109.5
N2A—C5A—H5A	108.4	H18E—C18B—H18F	109.5
C6A—C5A—H5A	108.4	O3A—C15A—H15D	109.5
C9A—C5A—H5A	108.4	O3A—C15A—H15E	109.5
C10B—C9B—C14B	119.3 (3)	H15D—C15A—H15E	109.5
C10B—C9B—C5B	121.5 (3)	O3A—C15A—H15F	109.5
C14B—C9B—C5B	119.2 (3)	H15D—C15A—H15F	109.5
C17B—C19B—C20B	133.4 (3)	H15E—C15A—H15F	109.5
C17B—C19B—S1B	112.9 (3)	C3B—O2B—C8B	116.9 (3)
C20B—C19B—S1B	113.6 (2)	O4A—C16A—H16D	109.5
C7A—C6A—C3A	120.8 (3)	O4A—C16A—H16E	109.5
C7A—C6A—C5A	122.6 (3)	H16D—C16A—H16E	109.5
C3A—C6A—C5A	116.5 (3)	O4A—C16A—H16F	109.5
N1A—C2A—N2A	122.3 (3)	H16D—C16A—H16F	109.5
N1A—C2A—S1A	124.5 (2)	H16E—C16A—H16F	109.5
N2A—C2A—S1A	113.2 (2)	C20A—C21A—H21D	109.5
C20B—C21B—H21A	109.5	C20A—C21A—H21E	109.5
C20B—C21B—H21B	109.5	H21D—C21A—H21E	109.5
H21A—C21B—H21B	109.5	C20A—C21A—H21F	109.5
C20B—C21B—H21C	109.5	H21D—C21A—H21F	109.5
H21A—C21B—H21C	109.5	H21E—C21A—H21F	109.5
H21B—C21B—H21C	109.5	O2A—C8A—C4A	105.8 (3)
C13B—C14B—C9B	120.2 (3)	O2A—C8A—H8A1	110.6
C13B—C14B—H14B	119.9	C4A—C8A—H8A1	110.6
C9B—C14B—H14B	119.9	O2A—C8A—H8A2	110.6
O3A—C12A—C11A	124.6 (3)	C4A—C8A—H8A2	110.6
O3A—C12A—C13A	115.0 (3)	H8A1—C8A—H8A2	108.7
C11A—C12A—C13A	120.4 (3)	C8A—C4A—H4A1	109.5
O4A—C13A—C14A	124.7 (3)	C8A—C4A—H4A2	109.5
O4A—C13A—C12A	115.2 (3)	H4A1—C4A—H4A2	109.5
C14A—C13A—C12A	120.0 (3)	C8A—C4A—H4A3	109.5
O3B—C12B—C11B	125.3 (3)	H4A1—C4A—H4A3	109.5
O3B—C12B—C13B	114.9 (3)	H4A2—C4A—H4A3	109.5
C11B—C12B—C13B	119.7 (3)	O1B—C3B—O2B	123.3 (4)
C19A—C17A—N2A	111.1 (3)	O1B—C3B—C6B	126.1 (4)
C19A—C17A—C18A	129.0 (3)	O2B—C3B—C6B	110.5 (3)
N2A—C17A—C18A	119.8 (3)	C4B'—C8B—C4B	43.9 (13)
O5A—C20A—C19A	117.3 (3)	C4B'—C8B—O2B	117.6 (8)
O5A—C20A—C21A	122.3 (3)	C4B—C8B—O2B	110.9 (4)
C19A—C20A—C21A	120.3 (3)	C4B'—C8B—H8B1	66.5
C6A—C7A—N1A	119.4 (3)	C4B—C8B—H8B1	109.5
C6A—C7A—C1A	128.0 (3)	O2B—C8B—H8B1	109.5
N1A—C7A—C1A	112.6 (3)	C4B'—C8B—H8B2	131.7
C9B—C10B—C11B	120.5 (3)	C4B—C8B—H8B2	109.5
C9B—C10B—H10B	119.8	O2B—C8B—H8B2	109.5
C11B—C10B—H10B	119.8	H8B1—C8B—H8B2	108.0
N2B—C5B—C6B	108.0 (3)	C8B—C4B—H4B1	109.5
N2B—C5B—C9B	109.2 (2)	C8B—C4B—H4B2	109.5
C6B—C5B—C9B	113.9 (3)	C8B—C4B—H4B3	109.5
N2B—C5B—H5B	108.5	C8B—C4B'—H4B4	109.5
C6B—C5B—H5B	108.5	C8B—C4B'—H4B5	109.5
C9B—C5B—H5B	108.5	H4B4—C4B'—H4B5	109.5
O5B—C20B—C19B	116.9 (3)	C8B—C4B'—H4B6	109.5
O5B—C20B—C21B	122.5 (3)	H4B4—C4B'—H4B6	109.5
C19B—C20B—C21B	120.6 (3)	H4B5—C4B'—H4B6	109.5
O1A—C3A—O2A	123.0 (3)		
			
C10A—C9A—C14A—C13A	−0.1 (5)	O4B—C13B—C12B—O3B	−2.2 (4)
C5A—C9A—C14A—C13A	−179.8 (3)	C14B—C13B—C12B—O3B	178.0 (3)
C16B—O4B—C13B—C14B	7.6 (4)	O4B—C13B—C12B—C11B	179.1 (3)
C16B—O4B—C13B—C12B	−172.3 (3)	C14B—C13B—C12B—C11B	−0.8 (5)
C14A—C9A—C10A—C11A	1.2 (5)	C2A—N2A—C17A—C19A	−6.3 (4)
C5A—C9A—C10A—C11A	−179.1 (3)	C5A—N2A—C17A—C19A	166.5 (3)
C12A—C11A—C10A—C9A	−0.3 (5)	C2A—N2A—C17A—C18A	170.4 (3)
C7B—N1B—C2B—N2B	−9.0 (5)	C5A—N2A—C17A—C18A	−16.7 (5)
C7B—N1B—C2B—S1B	168.6 (2)	C3A—C6A—C7A—N1A	−179.4 (3)
C17B—N2B—C2B—N1B	176.7 (3)	C5A—C6A—C7A—N1A	−2.3 (5)
C5B—N2B—C2B—N1B	−14.4 (5)	C3A—C6A—C7A—C1A	1.8 (5)
C17B—N2B—C2B—S1B	−1.1 (4)	C5A—C6A—C7A—C1A	178.9 (3)
C5B—N2B—C2B—S1B	167.7 (2)	C2A—N1A—C7A—C6A	−10.8 (5)
C19B—S1B—C2B—N1B	−178.0 (3)	C2A—N1A—C7A—C1A	168.1 (3)
C19B—S1B—C2B—N2B	−0.2 (3)	C14B—C9B—C10B—C11B	−0.6 (5)
C2B—N2B—C17B—C19B	2.3 (4)	C5B—C9B—C10B—C11B	−179.5 (3)
C5B—N2B—C17B—C19B	−166.1 (3)	C12B—C11B—C10B—C9B	−0.5 (5)
C2B—N2B—C17B—C18B	−175.7 (3)	C2B—N2B—C5B—C6B	28.3 (4)
C5B—N2B—C17B—C18B	15.9 (4)	C17B—N2B—C5B—C6B	−164.0 (3)
C2A—N2A—C5A—C6A	−22.7 (4)	C2B—N2B—C5B—C9B	−96.1 (3)
C17A—N2A—C5A—C6A	164.9 (3)	C17B—N2B—C5B—C9B	71.6 (4)
C2A—N2A—C5A—C9A	101.4 (3)	C10B—C9B—C5B—N2B	70.5 (4)
C17A—N2A—C5A—C9A	−70.9 (4)	C14B—C9B—C5B—N2B	−108.3 (3)
C10A—C9A—C5A—N2A	−70.4 (4)	C10B—C9B—C5B—C6B	−50.4 (4)
C14A—C9A—C5A—N2A	109.3 (3)	C14B—C9B—C5B—C6B	130.8 (3)
C10A—C9A—C5A—C6A	51.4 (4)	C17B—C19B—C20B—O5B	171.4 (3)
C14A—C9A—C5A—C6A	−128.9 (3)	S1B—C19B—C20B—O5B	−5.7 (4)
N2B—C17B—C19B—C20B	−179.4 (3)	C17B—C19B—C20B—C21B	−9.5 (6)
C18B—C17B—C19B—C20B	−1.6 (6)	S1B—C19B—C20B—C21B	173.4 (2)
N2B—C17B—C19B—S1B	−2.4 (4)	C8A—O2A—C3A—O1A	1.1 (5)
C18B—C17B—C19B—S1B	175.4 (3)	C8A—O2A—C3A—C6A	−177.9 (3)
C2B—S1B—C19B—C17B	1.5 (3)	C7A—C6A—C3A—O1A	12.5 (5)
C2B—S1B—C19B—C20B	179.2 (3)	C5A—C6A—C3A—O1A	−164.8 (3)
N2A—C5A—C6A—C7A	17.6 (4)	C7A—C6A—C3A—O2A	−168.6 (3)
C9A—C5A—C6A—C7A	−104.3 (3)	C5A—C6A—C3A—O2A	14.1 (4)
N2A—C5A—C6A—C3A	−165.2 (3)	N2B—C5B—C6B—C7B	−23.1 (4)
C9A—C5A—C6A—C3A	72.9 (3)	C9B—C5B—C6B—C7B	98.5 (4)
C7A—N1A—C2A—N2A	5.6 (5)	N2B—C5B—C6B—C3B	156.1 (3)
C7A—N1A—C2A—S1A	−173.7 (2)	C9B—C5B—C6B—C3B	−82.4 (4)
C17A—N2A—C2A—N1A	−174.1 (3)	C3B—C6B—C7B—N1B	−175.3 (3)
C5A—N2A—C2A—N1A	12.8 (5)	C5B—C6B—C7B—N1B	3.8 (5)
C17A—N2A—C2A—S1A	5.3 (4)	C3B—C6B—C7B—C1B	2.9 (5)
C5A—N2A—C2A—S1A	−167.9 (2)	C5B—C6B—C7B—C1B	−178.0 (3)
C19A—S1A—C2A—N1A	177.2 (3)	C2B—N1B—C7B—C6B	14.3 (5)
C19A—S1A—C2A—N2A	−2.2 (3)	C2B—N1B—C7B—C1B	−164.2 (3)
O4B—C13B—C14B—C9B	179.8 (3)	N2A—C17A—C19A—C20A	−177.7 (3)
C12B—C13B—C14B—C9B	−0.3 (5)	C18A—C17A—C19A—C20A	5.9 (6)
C10B—C9B—C14B—C13B	1.0 (5)	N2A—C17A—C19A—S1A	4.6 (4)
C5B—C9B—C14B—C13B	179.9 (3)	C18A—C17A—C19A—S1A	−171.8 (3)
C15A—O3A—C12A—C11A	8.8 (5)	O5A—C20A—C19A—C17A	−178.0 (3)
C15A—O3A—C12A—C13A	−172.5 (3)	C21A—C20A—C19A—C17A	4.3 (6)
C10A—C11A—C12A—O3A	177.0 (3)	O5A—C20A—C19A—S1A	−0.3 (4)
C10A—C11A—C12A—C13A	−1.7 (5)	C21A—C20A—C19A—S1A	−178.1 (3)
C16A—O4A—C13A—C14A	−7.3 (5)	C2A—S1A—C19A—C17A	−1.5 (3)
C16A—O4A—C13A—C12A	174.0 (3)	C2A—S1A—C19A—C20A	−179.7 (3)
C9A—C14A—C13A—O4A	179.5 (3)	C3A—O2A—C8A—C4A	177.7 (3)
C9A—C14A—C13A—C12A	−1.9 (5)	C8B—O2B—C3B—O1B	3.4 (6)
O3A—C12A—C13A—O4A	2.8 (4)	C8B—O2B—C3B—C6B	−176.6 (3)
C11A—C12A—C13A—O4A	−178.5 (3)	C7B—C6B—C3B—O1B	−13.9 (6)
O3A—C12A—C13A—C14A	−176.0 (3)	C5B—C6B—C3B—O1B	166.9 (3)
C11A—C12A—C13A—C14A	2.8 (5)	C7B—C6B—C3B—O2B	166.1 (3)
C15B—O3B—C12B—C11B	−10.9 (4)	C5B—C6B—C3B—O2B	−13.0 (4)
C15B—O3B—C12B—C13B	170.4 (3)	C3B—O2B—C8B—C4B'	160 (2)
C10B—C11B—C12B—O3B	−177.4 (3)	C3B—O2B—C8B—C4B	112.4 (8)
C10B—C11B—C12B—C13B	1.2 (5)		

### Hydrogen-bond geometry (Å, º)

Cg3 is the centroid of the C9*B*–C14*B* ring.

**Table:**

*D*—H···*A*	*D*—H	H···*A*	*D*···*A*	*D*—H···*A*
N1*A*—H1*A*···Cl1*A*	0.88	2.20	2.993 (1)	151
N1*B*—H1*B*···Cl1*B*^i^	0.88	2.20	2.992 (2)	150
C11*A*—H11*A*···O5*B*^ii^	0.95	2.58	3.523 (1)	173
C15*B*—H15*C*···O5*A*^iii^	0.98	2.58	3.456 (6)	148
C21*A*—H21*D*···O3*B*^iii^	0.98	2.50	3.459 (6)	166
C4*A*—H4*A*2···*Cg*3	0.98	2.63	3.551 (2)	157

Symmetry codes: (i) *x*+1, *y*, *z*; (ii) *x*−1, *y*, *z*; (iii) −*x*+2, −*y*+1, −*z*.
