# Supplementary figures and images for: Crystal structure of 2-acetyl-5-(3,4-di­meth­oxy­phen­yl)-6-eth­oxy­carbonyl-3,7-dimethyl-5H-thia­zolo[3,2-a]pyrimidin-8-ium chloride
Source: Acta Crystallogr E Crystallogr Commun. 2015 Sep 17;71(Pt 10):o764–5. doi: 10.1107/S2056989015016229 (PMC4647379; doi:10.1107/S2056989015016229)

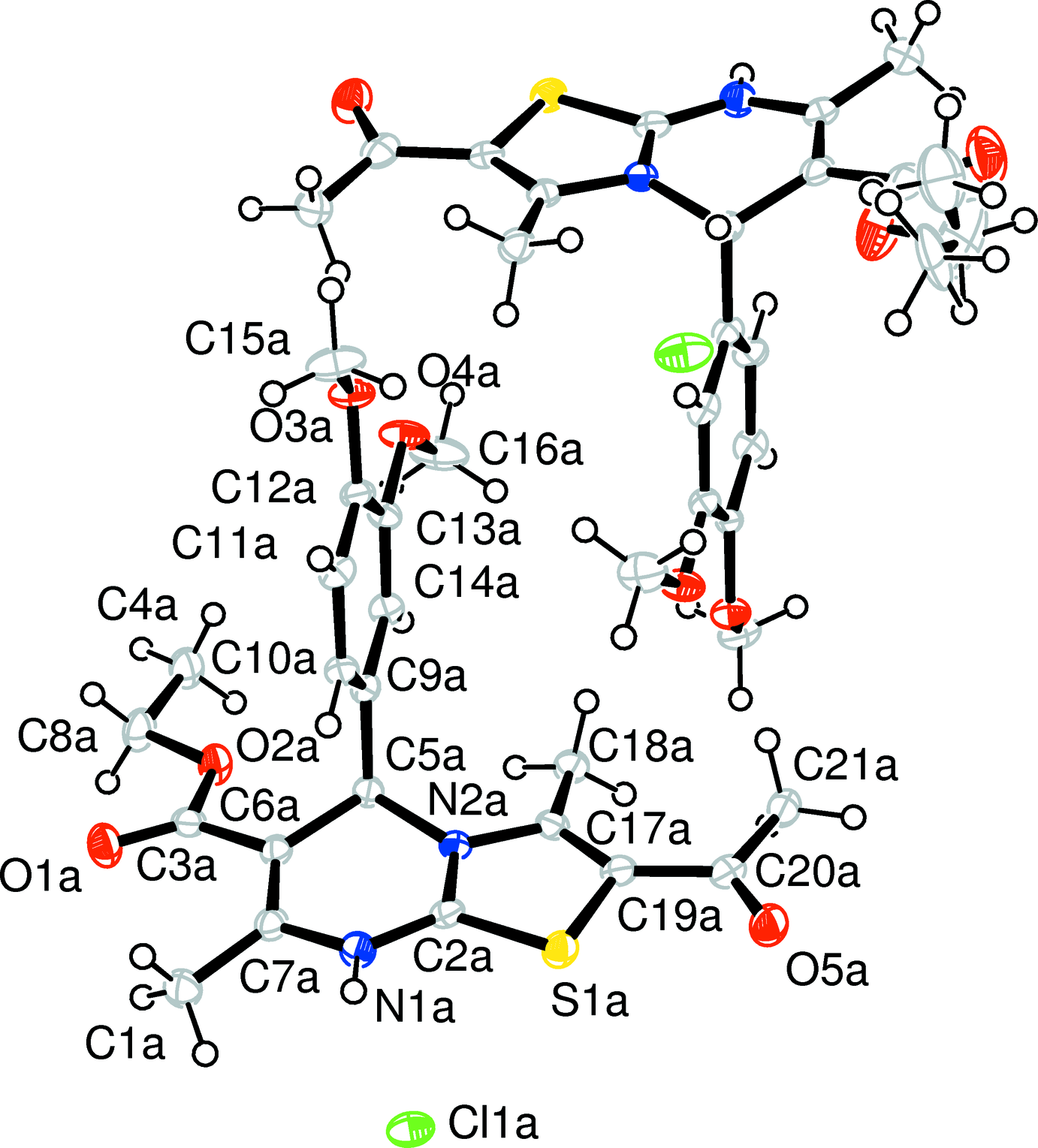

Supplement: Supplementary file 4 [file e-71-0o764-fig1.tif]

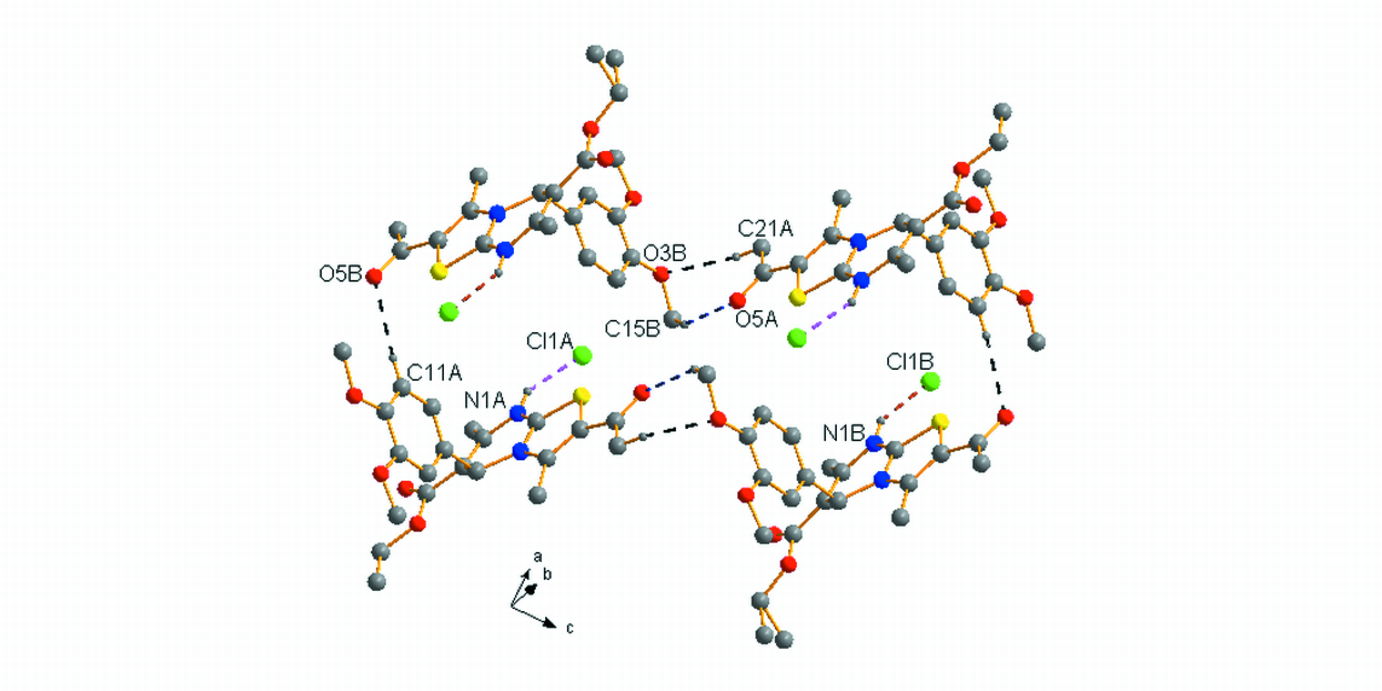

Supplement: Supplementary file 5 [file e-71-0o764-fig2.tif]

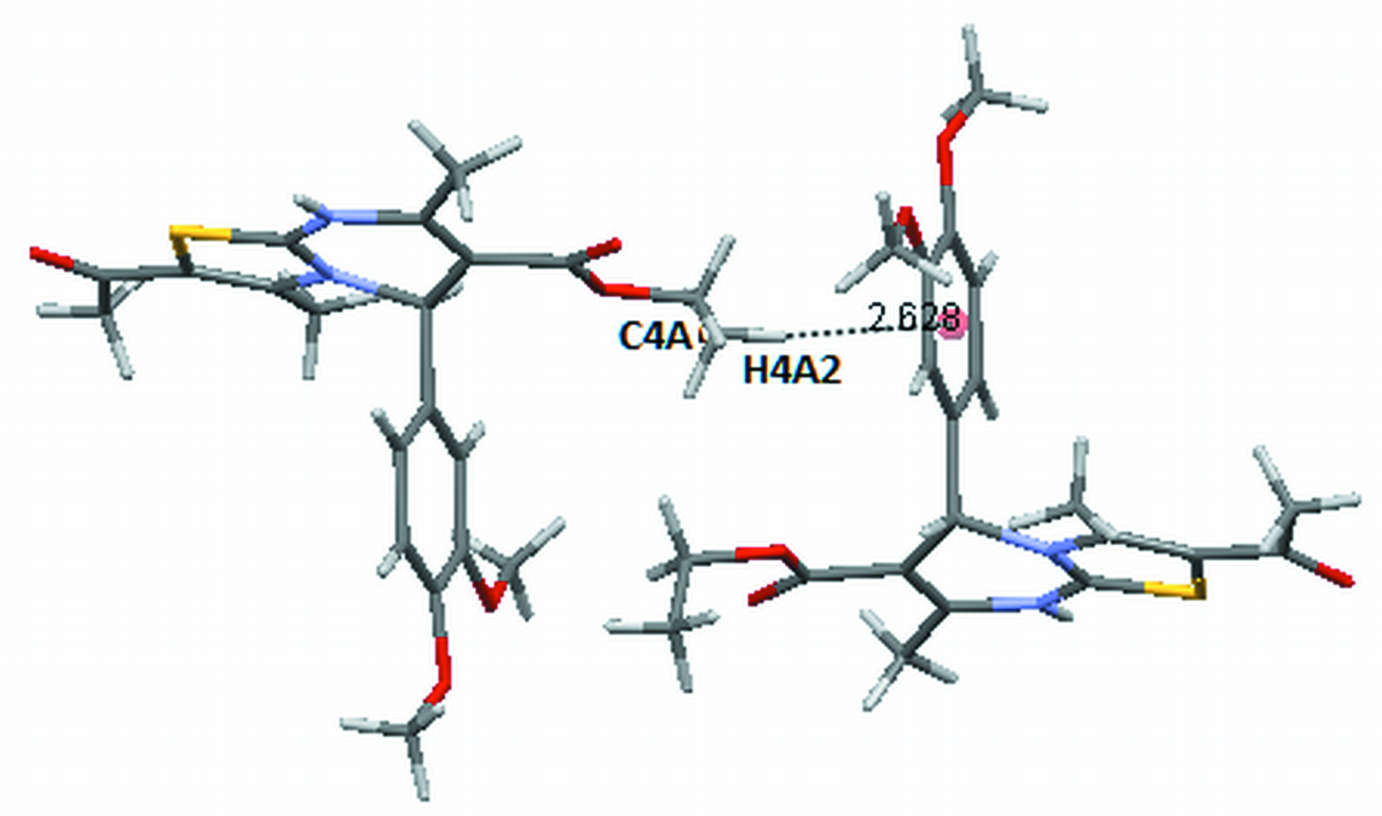

Supplement: Supplementary file 6 [file e-71-0o764-fig3.tif]
